# Translating three states of knowledge--discovery, invention, and innovation

**DOI:** 10.1186/1748-5908-5-9

**Published:** 2010-02-01

**Authors:** Joseph P Lane, Jennifer L Flagg

**Affiliations:** 1 School of Public Health and Health Professions, University at Buffalo (SUNY), Buffalo, NY, USA

## Abstract

**Background:**

Knowledge Translation (KT) has historically focused on the proper use of knowledge in healthcare delivery. A knowledge base has been created through empirical research and resides in scholarly literature. Some knowledge is amenable to direct application by stakeholders who are engaged during or after the research process, as shown by the Knowledge to Action (KTA) model. Other knowledge requires multiple transformations before achieving utility for end users. For example, conceptual knowledge generated through science or engineering may become embodied as a technology-based invention through development methods. The invention may then be integrated within an innovative device or service through production methods. To what extent is KT relevant to these transformations? How might the KTA model accommodate these additional development and production activities while preserving the KT concepts?

**Discussion:**

Stakeholders adopt and use knowledge that has perceived utility, such as a solution to a problem. Achieving a technology-based solution involves three methods that generate knowledge in three states, analogous to the three classic states of matter. Research activity generates discoveries that are intangible and highly malleable like a gas; development activity transforms discoveries into inventions that are moderately tangible yet still malleable like a liquid; and production activity transforms inventions into innovations that are tangible and immutable like a solid. The paper demonstrates how the KTA model can accommodate all three types of activity and address all three states of knowledge. Linking the three activities in one model also illustrates the importance of engaging the relevant stakeholders prior to initiating any knowledge-related activities.

**Summary:**

Science and engineering focused on technology-based devices or services change the state of knowledge through three successive activities. Achieving knowledge implementation requires methods that accommodate these three activities and knowledge states. Accomplishing beneficial societal impacts from technology-based knowledge involves the successful progression through all three activities, and the effective communication of each successive knowledge state to the relevant stakeholders. The KTA model appears suitable for structuring and linking these processes.

## Background

Knowledge translation (KT) represents a process for improving communication between the producers and consumers of knowledge to increase the application of research-based knowledge in practical forms. Moving knowledge into practice benefits a society by improving the quality of life for its members, and enhancing the economic competitiveness for its goods and services. The biomedical fields and medical professions initiated this KT movement [1,2]. They are able to analyze repositories of highly structured documentation on medical, surgical, and pharmacological interventions. Randomized controlled trials permit systematic reviews to establish evidence-based practices for consideration by stakeholders for the purpose of knowledge utilization. This is the thrust of the 'bench to bedside' initiatives in federally sponsored research programs [3].

The Canadian Institutes for Health Research (CIHR) has led efforts to structure the KT process [4]. Their Knowledge to Action (KTA) model describes how to match findings from completed research activity to the needs of knowledge users (*i.e*., end of grant KT), or by involving these stakeholders in ongoing research activity (*i.e*., integrated KT). It is important to note that the KTA model presumes a need to generate new knowledge and to do so through empirical methods.

### Knowledge Translation in technology-based rehabilitation science and engineering

The KT concept is now diffusing into other fields. Rehabilitation and the allied health professions are among the recent adopters of KT [5]. Rehabilitation is an applied human services context involving multiple medical, science, and engineering disciplines working in clinical, educational, vocational, or community settings. Their collective goal is to maximize the quality of life for persons with disabilities, regardless of their age, demographics, or diagnosis.

A person's functional status and goals drive the appropriate rehabilitation interventions. Functional impairments in a person's mobility, sensory systems, or cognitive abilities are viewed as gaps between the person's current capabilities and their optimal ability to perform desired activities. The field of rehabilitation employs clinical, home, or community-based interventions to restore, sustain, or supplement a person's functional capabilities. These rehabilitation interventions often involve technology-based devices or services. These devices and services were defined by Federal law in 1988 twenty years ago as 'assistive technology' [6].

The existence of assistive technology (AT) devices and services as interventions must be taken into account when considering how knowledge is translated and applied in the rehabilitation field. Publications from a major international KT conference recognized that the commercialization of technology-based devices and services represent a 'special case' of KT [7]. The commercialization process is far more complex than an exchange of conceptual knowledge between scholars, as it involves instrumental, conceptual and strategic use, the government, industrial and academic sectors, at least six stakeholder groups and three different methodologies. As Dr. Michael Gibbons stated in a KT keynote presentation:

'The once clear lines of demarcation between government, industry, and the universities, between science of the university and the technology of industry, between basic research, applied research, and product development, between careers in academe and those in industry no longer apply' [8].

From this perspective, no organization, investigator, or project is singularly responsible for completing the entire process of knowledge transformation. In fact, the concept of 'open innovation' is practiced by corporations to advance their interests through internal and external knowledge flows, and is equally relevant to knowledge exchanges between any source and their various stakeholders [9]. The government and academic sectors can facilitate the application of knowledge by embracing cross-sector collaboration via open innovation.

### Assumptions and definitions regarding knowledge

The KT literature notes that adopting new knowledge typically involves a measure of adaptation to fit the user's context [10]. For an applied field like rehabilitation and for the context of assistive technology devices and services, multiple stakeholders qualify as users, and some in turn become producers of knowledge in different forms for other users. The adoption of knowledge for technology-related projects clearly requires some adaptation of the assumptions and definitions underlying KT and its models. This article explores the feasibility of adapting the CIHR's KTA model in particular.

### Key assumption

Existing KT models are predicated on the goal of putting knowledge generated through academic research into practice. The application of research-based knowledge is expected to help solve a problem. A recent thematic analysis if 28 KT models [11] substantiated the focus on knowledge creation through research methods. These KT models--including the KTA model--represent knowledge creation and application as some form of academic research activity either underway or completed. With that assumption in place, the KTA model suggests one can either involve stakeholders after research activity is completed (end of grant KT), or involve stakeholders during the design and conduct of the research activity (integrated KT).

Knowledge Translation models and methods treat knowledge as existing in one state. This is the intangible conceptual state captured in the peer-reviewed literature generated by research activity conducted in the academic sector. However, knowledge exists in other states and may require transformation into other states to enable uptake and use by stakeholders. Knowledge in applied fields, such as those developing and producing technology-based devices and services, should be defined in a broader manner to include the various states of knowledge.

And just who are the stakeholders in the commercialization of technology-related knowledge? As one example, rehabilitation professionals involved with AT commercialization may collaborate with six different stakeholder groups:

1. Scholars who cite and integrate prior research findings in new studies;

2. Clinicians who recommend assistive technology to clients;

3. Consumers who apply personal experience when seeking AT;

4. Manufactures who participate in the design and critique of AT;

5. Resource Brokers who permit the adoption of new AT, or recommend intellectual property protection;

6. Policy Makers who set third-party reimbursement levels, or establish parameters of sponsored research programs [12].

### Implementing technology-related knowledge to solve problems

When knowledge is translated into action, the state of knowledge itself is transformed and it is important to ask: What are the knowledge states arising in this transformation process, and can KT accommodate those other states within its models?

Not all solutions to problems require the creation of new knowledge through research; nor does the direct application of conceptual knowledge always solve a problem. This is particularly true for technology-related knowledge that is defined by the application of knowledge in a tangible form. Funding agencies and investigators alike expect any technology-related solution to a problem to involve embodying knowledge in a tangible form.

Instances where existing technology cannot provide the desired function may prompt research activity to discover new capabilities. Or they may prompt a search for relevant discoveries from prior research that are extant in the literature. Such existing technology-related knowledge may be applied to solve a problem using methods other than research. For example, a project may employ development methods to transform conceptual knowledge into a tangible form--a prototype that proves that a conceptual application is feasible in a practical form. As another example, a project may employ production methods to transform the 'proof of concept' prototype into a device or service ready for application and use in the commercial marketplace. These technology development, transfer, and commercialization activities are not research, but instead are successive transformations of the research knowledge into other states. Their relevance to health and quality of life require expanding the underlying definition of knowledge. By differentiating the various states of knowledge that arise during the transformation process, KT may be able to accommodate methods beyond research within its models. This expansion and accommodation will help KT meet its goal of providing more effective technology-based health services and products [13].

### Three states of knowledge

Three methods of activity generate three different states of knowledge. Research activity generates knowledge in one state, while development activity and production activity generate knowledge in different states. The three states of knowledge represent a progression with the former states necessary for the latter to exist. The concept of open innovation recognizes the necessity of inter-sector collaboration in accomplishing the full range of transformations, with each state of knowledge dependent on the others.

The three states of knowledge are analogous to the three classic states of matter. This analogy will help clarify why the implementation of science in practice remains a challenging issue. Classically speaking, matter exists as gas, liquid, or solid (although plasma and a dozen additional states are now known). The three analogous states of knowledge are as follows.

### Discovery State of Knowledge

The technology-based solution to a specific problem may require the creation of new knowledge. Once a gap in knowledge is identified, the new knowledge can be recognized as a 'discovery.' A key attribute of a discovery is novelty, because it is the first articulation of something not previously known or demonstrated. Discoveries depend upon the scientific method to ensure validity and reliability. Despite presumed objectivity, their novelty may generate resistance if they contradict widely held beliefs [14]. Consequently, discoveries must be documented in a manner that permits independent replication. Lacking tangible form, discoveries are described in detailed manuscripts, which are submitted for peer-review for quality assurance. Those deemed valid are accepted for dissemination through journal articles or conference presentations. The publication system ensures the discovery is documented, attributed, and indexed for reference by others as a contribution to the global knowledge base. Publication ensures public disclosure and passively promotes awareness and use among stakeholders. Discoveries are malleable, subject to revision, rejection, or dispersion. As such, research-based discoveries are analogous to the gas state of matter.

### Invention State of Knowledge

Conceptual discoveries may become embodied in a tangible, yet provisional form--a proof of the concept's viability [15]. This second state of knowledge is called invention. An invention is something not previously demonstrated to be possible in practice. A key attribute of invention is feasibility. Feasibility combines with novelty; however, the invention and discovery do not have to occur together. One may apply independent prior discoveries to test the feasibility of a technology-based solution. This state change from discovery to invention requires the use of development models and methods that are distinct from those of research. Of course, the two activities may operate in tandem as suggested by the phrase 'research and development.' The output from this development activity is a proof-of-concept prototype. The prototype is a work in progress--a patchwork of elements, components, and external support systems, all combined to demonstrate feasibility. The demonstration of feasibility suggests potential functional applications that form the basis for intellectual property claims through the patenting process. The inventions are more tangible than discoveries, just as liquids are more tangible than gases, although inventions may still be shaped or formed in many different ways.

### Innovation State of Knowledge

Inventions may be further refined until they reach some final form, such as a functional device or service, capable of mass production, distribution, and support. This refinement is done with commercial intent, which is a perspective that academics are not trained to embrace. Dr. Chesbrough clearly defines this separate state:

'By innovation I mean something quite different from invention. To me innovation means invention implemented and taken to market.' [9]

The key attribute of knowledge embodied as an innovation is utility, in addition to the novelty and feasibility of the prior knowledge states. A technology-based solution may be feasible and novel in a laboratory setting, but utility is only achieved when the solution addresses the economic and operational constraints of the target user's problem in the context of the marketplace. Market utility means something of value, which is available to society in a consumable form. Transforming a prototype invention into an innovation requires yet another set of models and methods--those of new product development. Production methods ensure that the innovations final form is designed to meet constraints of functionality, physical dimensions, and cost. Accomplishing production activity requires a precise understanding of the intended market and the requirements of the customers for that device or service. The final form must be specified in exacting detail, as the raw materials and components must be ordered in economically advantageous quantities, while the tooling and assembly work must be planned to operate efficiently. Only then will the device or service be competitive in the commercial marketplace. The high level of specification and planning locks the innovation in a final form that can no longer be modified without substantial cost in materials and tooling. The innovation state of knowledge is equivalent to the solid state of matter. An innovation remains in the marketplace until replaced by another innovation offering greater utility. Such a replacement will have recapitulated the same sequential transformation of technology-related knowledge from research discovery, through development invention, and on out to production innovation.

### Three states of knowledge and KTA model

**Figure 1:**
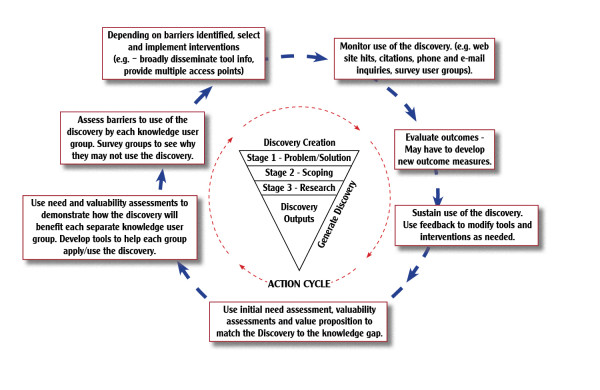
**Discovery Outputs**.

**Figure 2:**
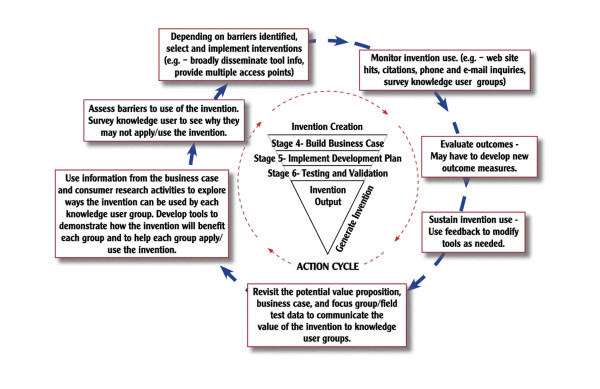
**Invention Outputs**.

**Figure 3:**
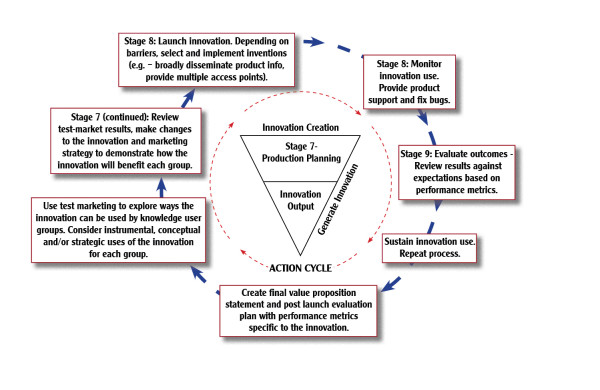
**Innovation Outputs**.

Differentiating between research-based discoveries, development-based inventions, and production-based innovations is a critical first step to generating operational versions of the KTA model pertaining to the context of technology transfer and commercialization. In fact, a study describing an operational version of the KTA model [16] gave rise to the idea of modifying the KTA model to accommodate the development and production phases of commercialization (see Figures 1, 2, and 3).

Specifically, the KTA's knowledge creation funnel representing research activity can be replicated to incorporate the development and production activities necessary to achieve invention and innovation outputs. Similarly, the KTA model's action cycle can be replicated to represent the different approaches necessary to effectively communicate the unique nature of discoveries, inventions, and innovations.

Adapting models is one thing. Ensuring fidelity to the concepts underlying the model is something else. The extant literature coupled with new research activity form the foundation for KT. These primary and secondary resources fuel the KT processes of quality assessment (rigor), synthesis (evidence), and tailored communication (relevance). What are the corollary concepts for technology-related projects? Rigorous quality assessments rely on the three methodologies (research, development, and production), each applied within their own context. Given the narrow focus of the eventual goal, decision making relies on the synthesis of primary evidence collected from the full range of stakeholders. Relevance is paramount for knowledge input and output, again focused on the eventual goal of a device or service in the marketplace.

The context of technology-related rehabilitation devices and services, has now adapted the assumptions and descriptions underlying the KTA model in the following ways: solving problems may involve technology-related knowledge drawn from the states of discovery, invention, and/or innovation; discovery represents novelty, invention requires both novelty and feasibility, while innovation embodies novelty, feasibility, and utility; and modelling the research, development, and production phases of activity is necessary to adapt the concepts and processes KT for incorporation into technology-related practices.

'Implementation science' exists as a topic of discussion because the methods used to create new knowledge are not designed to facilitate effective communication to a range of stakeholders, nor are they intended to ensure actual use by these stakeholders in practice. The implementation of scientific findings requires additional efforts. Traditionally passive dissemination and utilization strategies are used for scholarship, with the primary audience being others academics who read the journals and who attend the conferences for their own professional advancement. The shared culture and language that facilitates communication within this relatively closed system acts as a barrier for communication to other stakeholders. KT ensures that the knowledge producer works with the knowledge consumers. With input from knowledge consumers, the knowledge producers appraise the quality of research outputs, synthesize the work with other relevant sources, and translate the source format and language describing the conceptual discovery into formats and language most appropriate for effective communication to the outside stakeholders [17,7].

Both techniques are expected to lead to the direct application of discoveries by stakeholders. For technology-related discoveries, stakeholder use may require further research activity to expand the discovery or development activity to generate inventions. Stakeholder use may even continue through production activity to generate innovations. These downstream outcomes create opportunities for knowledge in the innovation state to have beneficial impacts on the quality of life for end users. The KT approach has both costs and benefits to the investigator. It can increase the likelihood of achieving the intended outcomes and impacts, and accelerate the timeframes involved in doing so. It also exacts significant additional costs, including the commitment of additional time, effort, and resources on the part of the knowledge producer. This is not a role for which academics are traditionally trained or rewarded, but these costs are no more discretionary than those required to ensure rigor in the research process itself.

Federal agencies allocate funds to university-based scholars for the purpose of generating discoveries through research methods. However, many federal agencies also allocate funds to university and corporate laboratories to generate development-based inventions, and to manufacturers for production-based innovations relevant to the federal agency's mission. All parties recognize the value of transforming technology-related knowledge into devices and services.

For applied research fields, such as such as technology-based devices and services, it is important to look beyond the first state of knowledge--discovery. The subsequent states of invention and innovation help frame how knowledge can be applied to solve problems related to quality of life. Given their contributions to the desired impact, the downstream roles of development and production activity should be considered from the inception point of any technology-related project.

Recall that the KTA model assumes on-going or completed research activity as the starting point. Even this point is fairly far along in the process. Before one can initiate research an agency identified a priority, wrote and circulated a request for proposals, applicants wrote and submitted proposals, a peer-review process occurred, and funding was awarded and disbursed according to some timeframe. Only then does research activity commence via project implementation. The stakeholders involved in these prior actions have done much to pre-ordain the problem as amenable to research-based knowledge applied by stakeholders.

### Need To Knowledge (NTK) model

By suspending the inherent assumption that the discovery outputs of research activity are the only outputs in need of translation, stakeholders are freed to consider how to solve problems with technology-related knowledge in the form of invention or innovation outputs. Six approaches to solving problems have been developed using various combinations of research, development, and production activities. It is important to note that quality appraisal and synthesis activities, which are key components of many KT models, are not described in these approaches. As portrayed in the discussion section of this paper, comparable activities are performed before research activity begins. Specifically, problem/solution definition carried out in collaboration with stakeholders and a series of preliminary assessments are designed to ensure rigor and relevance of the work. These steps obviate the need for additional quality appraisal and synthesis at the completion of research. Further, quality appraisal and synthesis activities occur throughout the NTK model using techniques appropriate for invention and innovation outputs.

### Six approaches to solving a problem with knowledge

1. Need to research to KT--Identify needs (problems) and potential solutions. Generate a new discovery (solution) and communicate its value to target stakeholders.

2. Need to research and development to KT--A new discovery, based on unmet needs, transformed into an invention, then offered to stakeholders for future innovation.

3. Need to research, development, and production to KT--A new discovery, based on unmet needs, transformed into an invention, and then specified as a device or service innovation, with its utility communicated to stakeholders.

4. Need to development and production to KT--An invention based on unmet needs and prior discoveries, transformed into an innovative device or service, with its utility communicated to stakeholders.

5. Need to production to KT--An innovation in the form of a device or service, based on unmet needs and prior research and development activity, distributed to stakeholders.

6. Need to KT--All the necessary research, development, and production work has already been done based on defined unmet needs. This option revisits the communication of the completed work to ensure it is offered in the appropriate forms and methods to the pertinent stakeholders for their future implementation.

Regardless of the chosen approach, all projects should integrate KT activities into their processes from their inception--a 'prior to grant' approach, rather than an end of grant or integrated approach to KT. As demonstrated in the preceding approaches, a 'prior to grant' approach starts with a defined need, such as a societal problem deemed worthy of government intervention. Appropriate due diligence then verifies that technology-related knowledge could solve the problem. Integration of stakeholders into the definition of problems and solutions ensures that future outputs in the form of discoveries, inventions, or innovations would have receptive stakeholders who are aware and ready for implementation. Using predefined needs to determine what knowledge to produce is the foundation of and reason for the title of the Need to Knowledge (NTK) model. This model does not assume that knowledge exists and must be put into action, but rather that needs exist, and knowledge may contribute to a solution.

If a funding agency requires projects to achieve fairly specific deliverables, a principal investigator could propose a scope that is bounded at the front end by any preceding activity as foundational knowledge, and bounded at the back end by ensuing activity to complete the continuum from problem input to solution impact. Any relevant prior research discoveries would find immediate application in ensuing development and/or production activities. Any ongoing research discoveries could be applied to the specific problem under study, while still being incorporated as contributions to the global knowledge base.

### Novel method of addressing current problem

The authors generated an operational KT model by expanding the KTA model's framework to integrate the three states of knowledge and the methods used to transform knowledge from one state to another. Each state of knowledge involves its own unique set of adaptations to the KTA model, both down through the 'knowledge creation funnel,' and out around the 'action cycle.' Taken together, the three iterations comprise the Need to Knowledge (NTK) model. The following section describes the key elements of the NTK model's structure in terms of stages, gates and steps.

## Discussion

### The Need to Knowledge (NTK) model

A 'prior to grant' perspective does not presume a requirement for research activity. Instead, it presumes that the application of technology-related knowledge in some state and through some activity may be a valid solution to a social problem. Thus, the definition of the need precedes the validation of a knowledge-based solution. The solution is expected to take the form of a technology-based device or service available to stakeholders in the marketplace. The solution follows from the problem definition. The NTK model expands the application of the KTA model from an exclusive focus on research methods to considering the methods most appropriate to solving the problem. For technology-related knowledge these include the methods applied in device or service development and those of industrial or commercial production. The methods for knowledge application and knowledge implementation deserve parity with the empirical methods for knowledge generation - at least within the applied contexts referenced here.

The NTK model represents the entire continuum of required activities, from problem statement through solution delivery. These activities are expected to be accomplished by some combination of stakeholders over time. Although presented here as a linear model, the collective activities may be recursive, iterative, or even disjointed. In this example, the model is applied to assistive technology for persons with disabilities. It may be equally applicable to all forms of technology-related innovations in fields such as medical, consumer products, housing, transportation, and alternative energy.

As previously described, the NTK model contains three phases, each named for the state of knowledge generated by the primary activity in that phase: discovery, invention, and innovation.

The three phases are cumulative in that successive knowledge states arise out of the preceding states. Iterations are possible. Invention state knowledge may reveal a need for additional discovery state knowledge. However, a project must stay focused on the goal, and not be drawn into a discovery/invention loop. The project's knowledge must progress to the innovations state to achieve the intended beneficial impact on a target audience.

Each phase contains three activity stages and three associated decision gates. The activity stages specify what the project needs to accomplish at that point. Some of the activities help the project progress sequentially. Other activities help the project prepare to address barriers encountered later in the process, or to obviate those downstream barriers entirely. KT recognizes the importance of tailoring the knowledge message to the language, culture, and values of each stakeholder group. The KT process itself can be tailored to the current knowledge state.

In the NTK model, each phase of activity ends with the subject knowledge in a different state than when the phase began. At the end of each phase, the project conducts KT activities tailored to that state of knowledge. The project should ensure that any knowledge is disclosed properly and with forethought for the subsequent consequences. KT is an opportunity to initiate active communication with the appropriate stakeholders regarding discoveries, inventions, or innovations, even while project work continues. In cases where the project terminates at the earlier knowledge states of discovery or invention, the KT process is a means for engaging stakeholders. This can be done by identifying lessons learned, sharing results from preliminary assessments and other forms of synthesis, such as a business case or technical report, and recommending opportunities for future endeavors. The stakeholders' experience may be more appropriate to continue the project through related methods to achieve the intended beneficial impact. Offering the aforementioned information in formats readily absorbed by the stakeholder group helps to ensure that the project will indeed move forward.

The NTK model is predicated on the three different states of knowledge involved in a technology-related project. An operational-level model needs to explicitly address these differences to ensure that the subject knowledge is effectively communicated to the relevant stakeholder groups, as it is successively transformed into different states. The following narrative explains how KT can be implemented within the NTK model.

### NTK Phase I. Discovery

Phase I conducts research activity to achieve the discovery state of knowledge. It involves three stages and three decision gates. Figure 1 adapts the KTA model to show the NTK model's discovery phase. It shows stages one, two, and three in the discovery creation funnel, and shows the appropriate activities to communicate a research-based discovery in the action cycle:

Stage one: Define problem and solution/gate one. Initiate project scoping?

Stage two: Project Scoping/gate two. Need for research-based discovery?

Stage three: Conduct research to generate discovery/gate three. Justification to generate a business case?

The CIHR's KTA model was designed for use with extramurally funded ongoing or concluded research projects. The KTA model may proceed from knowledge creation to problem application, or proceed from problem identification to knowledge creation. This is entirely appropriate for a model accommodating both inquiry- and need-driven research. The KTA model accommodates unanticipated or serendipitous opportunities to create and apply research.

In contrast, the NTK model contends that when both the sponsor and the investigator intend to solve a problem with a technology-related solution, the process should begin with the definition of the problem and the solution in stage one, and the identification of the appropriate method for effective intervention in stage two. In these instances, stages one and two are critical to ensure that government agencies are funding technology-related projects with actual relevance to society, and to ensure that an investigator's efforts are focused to generate beneficial impacts downstream.

The NTK model's discovery phase starts with stage one. The problem is defined before any research is initiated or even considered as a viable solution. Stage one defines a problem, articulates solutions, and establishes the overall goal. Stage two defines the project's potential contribution to the overall goal. One might assume a problem exists and propose a reasonable solution, or have anecdotal information about a problem/solution set within some bounded context. Neither is sufficient to justify the investment of public funds in a protracted process of knowledge creation and application. Both funders and grantees should be confident that the due diligence was performed in stage two to ensure that the project is novel, can be accomplished, fits within prior and ensuing work, and has a high likelihood of generating beneficial impacts through technology-related devices or services.

If stages one and two define and justify a requirement to generate new knowledge through research, stage three commences to do so. This is a key point of intersection between the NTK model's discovery phase and the KTA model's knowledge creation process. At that point, both models are engaged in the creation of new knowledge (discovery) while considering its subsequent application. As both of these models transition from the knowledge creation process to the action cycle, and from the discovery phase to invention phase, they both address a problem with conceptual knowledge. The critical difference between the KTA and NTK models is that the preliminary work performed in the NTK model's stages one and two provide a validated context for the application of the knowledge. These stages obviate the search for a problem context by starting with a problem and then designing a project to generate or apply knowledge as a solution.

The NTK discovery phase adapts the descriptions in KTA action cycle blocks to fit this focused context by revising the text to fit the discovery state of knowledge. As the NTK discovery phase action cycle moves in a clockwise direction, the stage one and stage two work provides invaluable information for communicating the discovery to the target audience, as well as to the other stakeholders who have potential uses for the discovery.

Customizing the form and content of a vehicle for communicating a discovery to each stakeholder group is central to the KT process. The customizing includes the language, culture, and value systems of each group, as well as the organizational level targeted (*e.g*., individual, organization, sector) [18]. The customizing should also consider the three types of knowledge use that may be pursued by individual stakeholders (*e.g*., instrumental, conceptual, symbolic/strategic) [19].

Creating a framework at this level of detail is very important for projects expected to result in technology-related devices or services. To achieve success, most if not all of the various stakeholder groups must recognize the value in the underlying knowledge. Various groups may have more or less appreciation for each of the three states of knowledge, but in the end they all must demonstrate support for the project's goal. The level of support among the stakeholders is an important input for the decision-makers involved in the decision gates that follow each stage of activity. If they determine that one or more stakeholder groups will either ignore or actively oppose the new device or service, internal decision-makers may terminate the project, or external decision-makers may withhold additional support.

Getting a new device or service introduced into the marketplace requires that all nine decision gates result in a decision to proceed. Each decision to proceed only leads to the next decision gate, while decisions to terminate a project or simply cease involvement stop progress toward the goal, but still call for KT activity. The NTK discovery phase is foundational work. This foundation may be built from the identification of previous knowledge discoveries, or it may require the creation of new knowledge. Nevertheless, the foundation alone is not sufficient to achieve the goal. The NTK discovery phase only encompasses one-third of the total number of stages. Decision gate three following stage three is a very important decision to move from discovery to invention. This decision has tremendous implications for time, effort, and resources. The decision-makers in the sponsor and project organizations should also be mindful of the importance of shifting the project's primary methodology from research to development.

As stated earlier, the conduct of research activity is optional within the NTK model. Decision gate two determines if the project initiates stage three research activity. The analyses conducted in stages one and two may determine that a technology-related solution does not require the discovery of new knowledge. The knowledge may already reside in the published literature, in which case the project moves directly to knowledge application under development methods. Or, the knowledge may reside in application in another field of use. In that case, the tools of technology transfer may be appropriate to apply as part of the development process. In either case, if the solution to the problem does not require research activity, the project could move directly from decision gate two to stage four within the invention phase.

### NTK Phase II. Invention

Phase II conducts development activity to achieve the invention state of knowledge. Figure 2 again adapts the KTA model to show the NTK model's invention phase. Figure 2 shows stages four, five, and six in the invention creation funnel, and shows the appropriate activities to communicate a development-based invention in the action cycle:

Stage four: Build business case and plan development/gate four. Implement plan?

Stage five: Implement development plan/gate five. Proceed to testing?

Stage six: Testing and validation/gate six. Plan for production?

The conceptual technology-related discovery generated or identified in phase I can now be transformed into knowledge in the invention state. The invention phase represents knowledge as a tangible asset with value. The phrase 'intellectual property' recognizes knowledge as such an asset. The patent and trademark system exists to identify and protect ownership of any intellectual property. The patent review considers both novelty and feasibility--the two attributes we define here as representing the invention state of knowledge. Novelty was established during the discovery phase, and now the project demonstrates its feasibility by designing and testing the knowledge in a prototype form.

A patent provides the invention owner with the legal rights to practice its use in applications yet to be determined. Beyond the patent reviewer's subjective decision that the invention is useful, the patent review process does not consider the objective market utility of the invention. This limitation supports this paper's distinction between an invention that must have a 'useful purpose' and be operational [20], and an innovation that must have commercial viability. For this reason, projects intended to result in an innovation must conduct preliminary work to verify not only the eventual utility of the intended device or service, but also its marketability. Stages four through six, described in the following paragraphs, ensure that these conditions are met.

Stage four, build business case and scope development plan, is a check to ensure that the next block of effort will likely meet the requirements of external partners--particularly the manufacturers and service deliverers. Researchers are not trained to consider the economic consequences of their actions, but the business case requirement ensures that the appropriate knowledge is gathered, synthesized, and analyzed in consideration of the external stakeholder partners. With this analysis in place, the investigator and their funding source can make an informed decision to implement the development plan or pursue another line of activity (decision gate four).

Stage five, implement development plan, follows from a decision to proceed. Development implementation involves building models or components that perform in practice the function envisioned in concept. These early stage models are called 'alpha' prototypes, as they are the preliminary versions. The alpha prototypes or their components are subjected to trial and measurement for the purpose of further refinement. User input is gained through focus groups to identify both essential and optional features and functions. The alpha prototypes represent successive approximations of the envisioned device or service, culminating with the beta prototype.

The next decision (gate five) is whether or not the beta prototype shows sufficient promise as a future device or service to warrant more comprehensive testing and validation. A decision to proceed requires a commitment for additional investment. The data and insights gained from the alpha version's technical, market, and user assessments are considered high quality primary source information, as it was generated through standard development methods. This information is synthesized, along with the investor's own considerations and constraints, to help formulate a decision to stop or to proceed.

Stage six, testing and validation of a beta prototype, is not an *ad hoc *process. There are formal protocols designed to pass the scrutiny of independent agencies. The methods involve sufficient rigor to ensure that the results reflect the actual functional capabilities of the prototype. Given the focus on the goal, the testing may require adherence to government or industry standards. Knowledge in the discovery state is not subjected to such scrutiny, yet careful calibration of performance may be necessary to win participation by external stakeholders including clinicians, manufacturers, or policy makers. Testing may involve both laboratory and field settings. The laboratory testing is a variation of research activity. Formal testing may require access to skilled technicians, fairly expensive instrumentation, and perhaps even controlled conditions. Both laboratory and field testing will involve human subjects representing the likely or potential users of the device or service. The testing and validation typically reveals additional opportunities to refine and improve the prototype device, particularly through feedback obtained from human subjects. Additional testing may be required to confirm that any changes have not detracted from established performance parameters.

These three stages and their underlying steps apply development methodologies to build and test prototypes representing the intended technology-based device or service. This work is conducted within the framework of a business case, in recognition of the role of private sector manufacturers in the subsequent transformation. The stages and steps draw heavily from the standard practices established by industry for new product development. This ensures the process rigor and user relevance, along with the quality of evidence generated at each step. The Product Development Manager's Association (PDMA) has extensively described many of these practices in a series of reference publications [21,22].

Being mindful of the eventual goal for a device or service in the marketplace helps investigators--whether in academia or industry--make sound decisions in this interim invention phase that preserve the asset's future value to others. Development work that might satisfy intellectual interests as an end in itself, may not satisfy the requirements of external stakeholders who will be responsible for investing the time and resources to transform an invention into an innovation for the marketplace. The business case provides a template for defining the required development work, some of which may appear superfluous to those not trained to anticipate the downstream requirements of the innovation phase. The business case guides the investigator's allocation of time and resources, and ensures the results are relevant to the goal.

Even in technology-related fields, an investigator's efforts may not lead to an invention with commercial potential. There may be ancillary benefits that satisfy academic incentives, such as funding and publications, but these inputs and outputs are not the goal. A recent analysis of research and development activity within the field of rehabilitation engineering showed that most projects do not achieve the intended outcomes [23]. Most development projects that did not progress from invention to innovation had not adequately addressed the requirements of the external stakeholders on which the eventual outcome depended.

With the completion of stage six, testing and validation, the tangible device or service has progressed from alpha, through beta, and on to a pre-production prototype. If the investigator has not yet claimed the underlying intellectual property, this pre-production version provides all the details necessary. If a patent application was filed previously, it can be amended to include any refinements. The invention phase closes with one of two final actions. If the investigator's role had been set to end upon completion of the invention phase, the activities related to KT for knowledge in the invention state should be initiated.

However, if the investigator had planned to continue their involvement in the project throughout the innovation phase, then they must consider decision gate six, to go or not go forward to production planning. The testing and validation may have revealed new information regarding the viability of the product or service or its market potential, and the investigator must carefully consider their decision to either terminate or continue the project. In either case, they should initiate KT for the invention state output of the subject knowledge. This is a critical step because the investigator will likely need a corporate collaborator to implement the innovation phase. The knowledge generated through standard development methods, and organized within the framework of the evolving business plan, gives the external partner the right information in the right form for their consideration. To the extent the project investigator has practiced KT, a corporation can make a sound and informed decision regarding future involvement. It is better to enlist a partner that is committed for the long-term than to convince a partner in the short-term who decides to withdraw in the future.

The NTK invention phase represents a substantial increase in project expenditures (*i.e*., so-called 'sunk costs') that include the time, effort, and resources applied to the previous stages. In its embodied state as a proof of concept, the prototype is considered property with value as an asset. This pre-production form has assumed the knowledge state analogous to a liquid. It is less malleable than a discovery (gas) and more malleable than a finished product or service (solid). The translation process is different for knowledge in this liquid state, so the means, message, and method must be different from those used to communicate the discovery in its conceptual (gas) state.

The three stages (four through six) of the invention phase transform conceptual discoveries into embodied inventions. The action cycle works with knowledge in this more refined and less flexible state, so it begins with a more focused message to the relevant knowledge users. Depending on their roles, these stakeholders may be able to put knowledge about the prototype device or service directly into use, or they may be involved in the ensuing innovation phase of activity.

The invention phase is only the middle third of a triad of activity. If the gate six decision is to terminate the project, then widely disclosing the prototype might be the only option for generating stakeholder awareness. A decision to continue the project reaffirms the original goal of a new or improved technology-based device or service in the marketplace. In that case, the intellectual property must be protected as an asset, as well as protected from improper or untimely disclosure. The investigator and related stakeholders must balance the desire to communicate the invention, with the need to preserve the invention's value for the innovation phase. This is often where a conflict arises between academia's drive to publish and industry's drive to maintain secrecy.

### NTK Phase III. Innovation

Phase III conducts production activity to achieve the innovation state of knowledge. Figure 3 further adapts the KTA model to show the NTK model's innovation phase. Unlike Figures 1 and 2, the three stages and decision gates in the innovation phase are distributed across both the innovation creation funnel and the action cycle. This is because a successful device or service innovation requires continuous and iterative interactions between the producers and the consumers--between the investigators and the stakeholders:

Stage seven: Production planning and preparation/gate seven. Go to launch?

Stage eight: Launch innovation/gate eight. Shift from launch to maintenance?

Stage nine: Post-launch assessment/gate nine. Continue, terminate, replace?

The transformation from an invention state prototype to an innovation state device or service is not typically the domain of scholars. Scholars in the academic sector are trained and supported to generate discoveries through research methods. Executives in the industrial sector are trained and supported to generate innovations through production methods. Both scholars and executives lay partial claim to the shared territory of development, although the term has different meanings to each sector. Scholars speak of development in their academic context of refining a theory, testing a hypothesis, or generating additional evidence for a position. Executives speak of development in their production context, testing and validating pre-production prototypes and their underlying technology-based capabilities.

Some scholars do function as entrepreneurs or collaborate with industry as consultants, just as some executives participate in the academic process. These exceptions prove the rule of having experts lead in their areas of expertise. Accomplishing the project's goal is highly dependent on an external manufacturer's decision to collaborate in the innovation phase. Scholars do not produce and deliver devices or services to the marketplace, nor do policy makers or clinicians. The innovation phase is typically directed by executives working for manufacturers. In this third phase of the overall process, the executives base their decisions on the foundational work completed in the discovery and invention phases. The preparatory work in stages one through six needed to build a convincing argument for proceeding in terms that the manufacturer can understand and accurately value--a business case. After all, communicating effectively in language and formats best understood by the audience is a core attribute of KT.

In the hands of a qualified, competent, and financially sound corporation, the production planning and preparation proceeds smoothly. Such manufacturers are experienced in executing the great number of steps in the high level of detail involved. The innovation phase transforms the knowledge from a semi-malleable state to a solid state. In stage seven, the specifications created for tooling, materials, logistics, and support essentially 'freeze' the design into a form that can be replicated in great numbers at an affordable cost. These steps are detailed within the Product Development Managers Association (PDMA) materials on new product development, so they are not described here [21,22].

Even after all of the effort expended in stage seven, the project leaders need the discipline and objectivity to decide whether or not to introduce the device or service into the marketplace (decision gate seven). A private sector heuristic is to ignore the sunk cost--the go or no go decisions should be made without considering the prior investment. A project should cease if it does not look promising despite all of the prior efforts to demonstrate its worth. This decision requires a particular perspective based on two factors. First, these private sector decision-makers are stewards of resources belonging to the corporate entity or its shareholders. Recipients of government funding may not share that perspective. Second, private sector organizations typically have multiple projects so they can act without emotional or professional attachment to any one option. In contrast, recipients of government funding may be operating as independent investigators or as part of a small team, without options for expending the available resources. The latter may proceed with the project launch simply because there is no other option for expending the resources and supporting themselves in the process. Individual project managers in a corporation may advocate for their own projects but they are operating within a hierarchy.

The gate seven decision is typically made at the highest executive level by people who are best positioned to act in the interest of the corporation. This is not the same as acting in the best interest of society. The rationale for keeping many technology-related projects in the academic sector is that corporations lack the profit motive to participate. Unfortunately, those projects still need corporate buy-in to eventually become available in the marketplace. The NTK model's early interest in establishing the business case is based on this pragmatic situation. If the business case calls for government subsidy then that is an issue to be resolved sooner rather than later.

As shown in Figure 3, the stage seven activity begins in the innovation creation funnel but then continues on into the action cycle. The production methods require high levels of stakeholder interaction regarding test marketing to hone the form and content of messages used to communicate the innovation's objective utility to potential customers. The results of all of this limited release, test marketing and internal review lead to decision gate seven--go to launch?

A decision to proceed initiates stage eight, product launch. This entails a mass production process by the manufacturer. The accompanying marketing, promotion, and advertising are focused on the essence of KT--achieving stakeholder awareness, interest, adoption, and use of the device or service being promoted. The activity involved is widely understood due to the success of our mass marketing and media culture. Decision gate eight shifts efforts from launch to maintenance levels. A corporation cannot sustain the expenses involved in a launch indefinitely, and those efforts may artificially inflate evidence of awareness, interest, and use. Moving from launch to maintenance permits the corporation to consider the market viability of the device or service on its own merits.

Stage nine is the post-launch assessment. The corporation must now decide if the device or service is sustainable, and whether it should be integrated into its core product mix. This assessment continues for the innovation's life cycle as the device or service tracks through the marketplace's curve of introduction, growth, and maturation.

The assessment is not limited to the phase III innovation activity, but will likely involve a summative-level evaluation of the entire NTK model process. The assessment asks, 'how well did the project perform at accomplishing the goal?' The answer will feed into the decision gate nine, where a decision is made to terminate the production activity, or to repeat the entire three phase process to generate a new or improved version of the device or service. Even for successful products, manufacturers will eventually decide to repeat the entire process. They know that competing companies will create similar devices or services to compete for market share. Therefore, the best chance of staying ahead in such a competitive environment is to initiate work on the next generation device or service. This practice is known in industry as continuous quality improvement.

## Summary

The KT process moves knowledge into application. Existing KT models focus on knowledge as conceptual discoveries generated through research methods. However, projects intended to move technology-related knowledge into application apply two additional methods: development methods that transform conceptual discoveries into tangible inventions, and production methods that transform inventions into device or service innovations. These three states of knowledge outputs are described as analogous to the three classic states of matter: gas, liquid, and solid. The analogy suggests that transforming knowledge into each state, and then translating knowledge outputs from each state, must consider multiple methods.

The paper demonstrates how the widely cited KTA model can be adapted to accommodate all three states of knowledge. The resulting NTK model begins by identifying a problem (need) and then defining a technology-related solution (knowledge). This deliberately focused approach is necessary to ensure the novelty, feasibility, and utility of the eventual solution. The stage/gate model describes the progression through the three states of knowledge, and the KT activities most appropriate for communicating each knowledge state to the relevant stakeholders.

The NTK model is offered as an operational framework for technology-related projects, where the intended application requires these knowledge transformations to reach the marketplace as a device or service. Additional material related to this paper--including the NTK model in detailed electronic form--can be found at http://www.kt4tt.buffalo.edu.

The following summarizes the article's key points:

• Technology-related knowledge exists in three states analogous to the three states of matter: research discoveries are the gas state, development inventions are the liquid state, and production innovations are the solid state.

• Applying technology-related knowledge as solutions to societal problems requires careful consideration of the relevant state of knowledge in the project, and the methods applied to transform the knowledge from one state to the next.

• Knowledge translation models can be expanded to accommodate all three knowledge creation methods, and to effectively communicate all three states of knowledge to the target stakeholders.

• The resulting operational model may be applied to any project intending to create and apply technology-related innovations to benefit society.

## Competing interests

The authors declare that they have no competing interests.

## Authors' contributions

JPL organized the framework, conceived the links between knowledge translation and technology transfer, suggested the states of knowledge, and linked discovery, invention, and innovation in the model. JLF conducted a review of academic and industry literature and applied the results to the stages and steps within the creation and action segments of the three model phases. Both authors have read and approved the final version of this manuscript.
